# Pratique de l’échocardiographie transœsophagienne au Burkina Faso: analyse situationnelle et perspectives de développement

**DOI:** 10.11604/pamj.2015.21.157.5862

**Published:** 2015-06-24

**Authors:** Nobila Valentin Yaméogo, Larissa Justine Kagambèga, Georges Rosario Christian Millogo, Aminata Niankara, Koudougou Jonas Kologo, Boubacar Jean Yves Toguyéni, André Samadoulougou, Patrice Zabsonré

**Affiliations:** 1 Service de Cardiologie CHU-Yalgado Ouédraogo, Ouagadougou, Burkina Faso; 2 UFR Sciences de la Santé, Université de Ouagadougou, Ouagadougou, Burkina Faso

**Keywords:** ETO, indications, résultats, état des lieux, Burkina Faso, transesophageal ultrasound, indications, results, current situation, Burkina Faso

## Abstract

**Introduction:**

L’échocardiographie transœsophagienne (ETO) est une technique relativement récente mais elle est devenue un complément indispensable à l’écho Doppler cardiaque transthoracique dans certaines pathologies. Elle constitue encore un luxe dans certains pays en développement. Ce travail avait pour objectif de faire l’état des lieux de la réalisation de l'ETO dans notre pays.

**Méthodes:**

Nous avons réalisé une étude transversale descriptive qui s'est déroulée du 1^er^ mars 2011 au 31 juillet 2013 dans deux centres hospitaliers de la ville de Ouagadougou incluant les patients adressés pour ETO. L'examen a été réalisé par un cardiologue avec un échographe de marque Siemens muni d'une sonde d'ETO. Une surveillance de la tension artérielle et du rythme cardiaque était réalisée en cours d'examen et une mise en observation de 15 minutes était respectée après l'examen. L’épreuve de contraste était systématique en dehors des cas de communication interatrial évidente.

**Résultats:**

Durant la période de l’étude, 142 ETO ont été réalisées. L’âge moyen des patients était de 44,6 ± 11,9 ans. Le sex-ratio était de 1,3 en faveur des femmes. Les indications étaient dominées par les endocardites (19%), les accidents vasculaires cérébraux ischémiques (16,9%) et les évaluations de prothèses valvulaires (15,5%). L'introduction de la sonde était facile dans 97,2% des cas et laborieuse dans les autres cas. Aucun accident n’était mentionné. L'ETO était normale dans 28,2% des cas. Les résultats pathologiques étaient représentés par les masses intracardiaques (21,1%), les anomalies du septum interatrial (19%), les endocardites (13,4%) et les pathologies de l'aorte (10,6%).

**Conclusion:**

L'ETO est un examen maîtrisé dans notre pays mais reste encore à être développé. Ce développement passe par la formation du personnel et l’équipement en échographes munis de sondes d'ETO.

## Introduction

L’échocardiographie transœsophagienne (ETO) est une technique relativement récente [1]. Elle est en effet née en 1975 mais est rapidement devenue un complément indispensable à l’échographie cardiaque transthoracique dans plusieurs affections cardiovasculaires, comme les pathologies aortiques, les endocardites infectieuses, les pathologies valvulaires, la recherche de cardiopathies emboligènes, les dysfonctions de prothèse valvulaire et la surveillance per- et postopératoire [2]. Très développée dans les laboratoires d’échographie des pays développés, elle représente un luxe dans certains centres au sud du Sahara. Au Burkina Faso, elle est effective depuis 2011. Ce travail s’était donné pour objectif de faire l’état des lieux de la réalisation de l'ETO dans notre pays.

## Méthodes

Nous avons réalisé une étude transversale descriptive qui s'est déroulée du 1er mars 2011 au 31 juillet 2013 dans deux centres hospitaliers de la ville de Ouagadougou (le service de cardiologie du CHU-Yalgado Ouédraogo et la clinique médicale KABORE). Nous avons inclus de manière consécutive tous les patients adressés pour ETO dans les deux centres. Nous avons recherché la qualité du personnel demandeur, l’âge des patients, les indications et les résultats de l'examen. L'examen a été réalisé avec un échographe de marque Siemens muni d'une sonde d'ETO (CHU). L'examen était réalisé par un cardiologue sénior. La réalisation de l'ETT avant l'ETO était systématique. La prémédication était réalisée avec la xylocaine spray ou gel. Le jeûn était de 8 heures. Une explication détaillée et rassurante de la procédure était faite à l'endroit des patients. Les prothèses dentaires étaient retirées. Le patient était installé en décubitus latéral gauche tête fléchie, bouche ouverte. La sonde était introduite à travers un cale-dents. Une surveillance de la tension artérielle et du rythme cardiaque était réalisée en cours d'examen. Une mise en observation de 15 minutes après l'examen était observée et le repas autorisé au moins 2 heures après la fin de l'examen. Nous avons réalisé des coupes transœsophagiennes hautes et basses et transgastriques et avons recherché selon les indications de l'examen, les pathologies ou anomalies de l'aorte, du septum interatrial, de l'endocarde, de même que les masses et contrastes spontanés intracardiaques et auriculaires. L’épreuve de contraste était systématique en dehors des cas de communication interatrial évidente. Une mise en observation de 15 minutes était respectée après l'examen au cours de laquelle une surveillance de la tension artérielle et de la fréquence cardiaque était assurée.

## Résultats

Durant la période de l’étude, 142 ETO ont été réalisées dont 117 au CHU (82,4%) et 25 (17,6%) à la clinique KABORE. Tous nos patients étaient conscients. L'introduction de la sonde était réalisée en décubitus latéral gauche dans 95,6% des cas et en position assise dans les autres cas, après une prémédication par la Xylocaïne en Spray ou en Gel. Les prescripteurs étaient des cardiologues dans 79,6% des cas et des neurologues dans les autres cas. L’âge moyen des patients était de 44,6 ± 11,9 ans (extrêmes de 20 et 77 ans). Le sex-ratio était de 1,3 en faveur des femmes. Les indications étaient dominées par les endocardites (19%), les accidents vasculaires cérébraux ischémiques (16,9%) et les évaluations de prothèses valvulaires (15,5%). Les différentes indications sont résumées dans le Tableau 1. L'introduction de la sonde était facile dans 97,2% des cas et laborieuse dans les autres cas. Aucun accident n’était mentionné. Cependant, 12 cas d'incidents était survenus; il s'agissait de céphalées (5 cas), de vertiges (4 cas), de hausse de la tension artérielle (2 cas) et de fibrillation atriale (1 cas).

**Tableau 1:** Distribution des indications de 142 ETO réalisées dans la ville de Ouagadougou

Indications	Nombre	%
Endocardite	27	19
Accident vasculaire cérébral ischémique	24	16,9
Evaluation de prothèse valvulaire	22	15,5
Etude de valvulopathie	21	14,8
Communication interatriale	16	11,3
ASIA/FOP	14	9,9
Masse intracardiaque	12	8,5
Dilatation de l'aorte	4	2,8
Cardiopathie congénitale	4	2,8
Cardiopathie congénitale	3	2,1
Dissection aortique	3	2,1

### Résultats de l'ETO

L'examen était normal dans 28,2% des cas.

**Figure 1:**
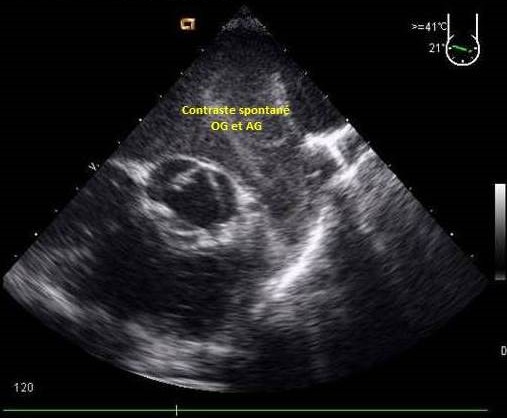
ETO coupe œsophagienne moyenne montrant un contraste spontané intraatrial (OG) et auriculaire gauche (AG)

**Figure 2:**
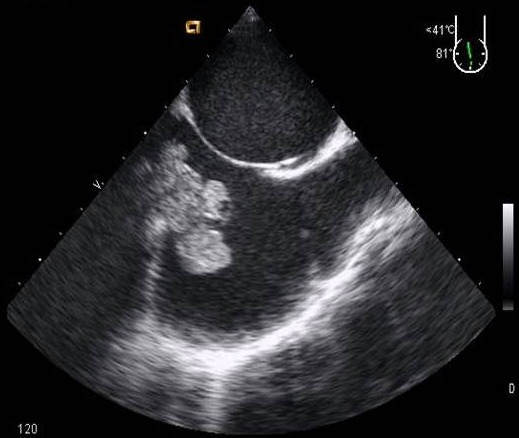
ETO coupe œsophagienne moyenne atriobicave objectivant une tumeur en grappe de raisin à l'abouchement de la veine cave inférieure

**Les masses intracardiaques et contraste spontané:** elles étaient retrouvées dans 21,1% des cas et constituées dans 93,3% de thrombus et contraste spontané (Figure 1) et dans 6,7% de tumeur (Figure 2).

**Figure 3:**
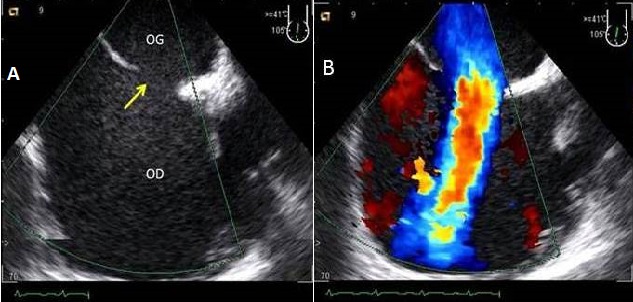
ETO coupe biatriale objectivant une large communication interatriale (flèche) 2D (A) et Doppler couleur montrant le flux de CIA

**Figure 4:**
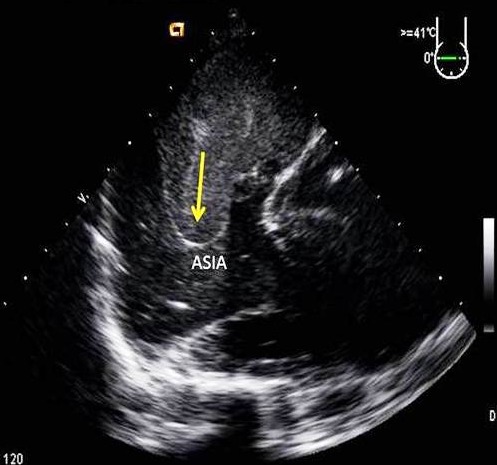
ETO œsophagienne moyenne. Anévrisme du septum interatrial (ASIA -flèche-) avec contraste spontané

**Les anomalies du septum interatrial:** les anomalies du septum interatrial étaient retrouvées dans 19% des cas, soit 44,4% de communication interatrial (Figure 3), 37% d'anévrisme du septum interatrial (Figure 4) et 18,5% de foramen ovale perméable.

**Figure 5:**
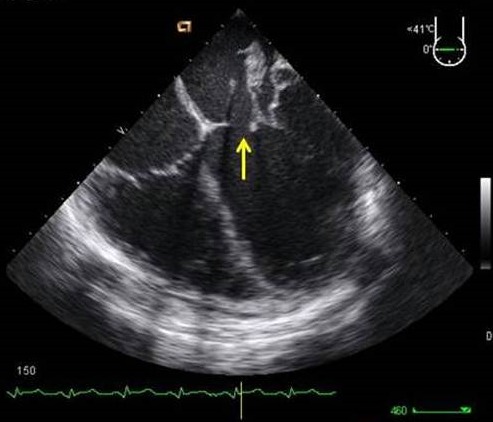
ETO coupe œsophagienne moyenne 0 degré mettant en évidence une endocardite mitrale avec mutilation de la valve septale qui est perforée (flèche)

**Les endocardites:** elles étaient retrouvées dans 13,4% des cas. Elles étaient de localisation mitrale dans 52,6% des cas des endocardites (Figure 5), aortique dans 26,3% cas et mitro-aortiques dans 21,1% des cas.

**Figure 6:**
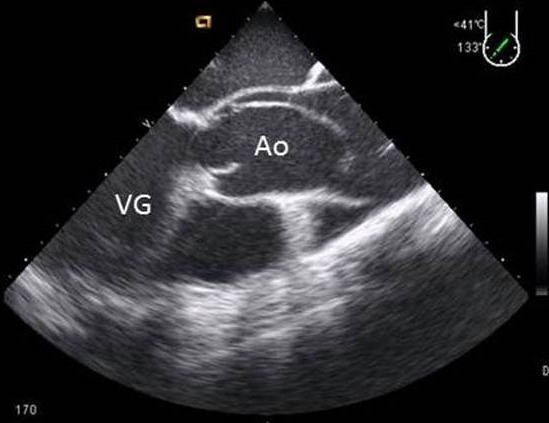
ETO coupe œsophagienne moyenne montrant dissection de l'aorte de type A

**Figure 7:**
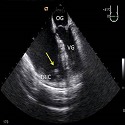
ETO coupe œsophagienne moyenne mettant en relief les cavités cardiaques droites. Insertion anormalement basse des valves tricuspide coorespondant une maladie d'Ebstein

**Pathologies de l'aorte:** elles représentaient 10,6% des résultats pathologiques. La dissection de l'aorte (Figure 6) représentait 20% de la pathologie aortique. Ailleurs, une maladie d'Ebstein était retrouvée dans 2 cas (Figure 7). Les résultats de l'ETO sont représentés dans le Tableau 2.

**Tableau 2:** Répartition des résultats de 142 ETO réalisées dans la ville de Ouagadougou

Résultat	Nombre	%
Normal	44	31
Thrombose intracardiaque cardiaque	28	19,7
Valvulopathie rhumatismale	21	14,8
Endocardite	19	13,4
Communication interatriale	12	5,9
Anévrisme du septum interatrial	10	8,5
Athérome aortique	6	4,2
Maladie annulo ectasiante de l'aorte	6	4,2
Foramen ovale perméable	5	3,5
Dysfonction de prothèse valvulaire	4	2,8
Dissection aortique	3	2,1
Cœur triatrial	2	1,4
Maladie d'Ebstein	2	1,4
Non compaction isolée du ventricule gauche	2	1,4
Tumeur intracardiaque	2	1,4

## Discussion

Cette étude montre que l'ETO est une technique bien maitrisée dans notre pays. En effet, même si notre échantillon semble de petite taille, nous n'avons enregistré aucun échec d'introduction de la sonde et aucun accident n'a été noté. Mais en pratique, l'examen est semi invasive et les accidents sont très rares. Dans l’étude monocentrique de James et al. [3], qui a porté sur 10 000 ETO réalisées en 10 ans, un cas de perforation de l'hypoharynx était noté (0,01%), 2 cas de perforation de l’‘sophage cervicale (0,02%) et aucune perforation gastrique de même qu'aucun décès n’étaient enregistrés. Certaines équipent rencontrent des accidents plus graves comme une double rupture de l'estomac enregistrée par une équipe de l'hôpital du Haut-Lévêque en France [4]. Ces différentes complications semblent plus fréquentes chez le sujet âgé surtout du genre féminin [3]. La supériorité de l'ETO par rapport à l'ETT ne souffre d'aucun doute, en témoigne nos résultats. Malheureusement, seuls deux échographistes sont formés et entraînés à la réalisation de cet examen. Il n'est disponible que dans un seul centre hospitalier universitaire des quatre que compte notre pays. Depuis ces dernières décennies, l'ETO est largement utilisée en cardiologie. Son recours croissant résulte de sa capacité en temps réel à évaluer la fonction myocardique, la performance valvulaire, les pathologies des gros troncs de la base, les cardioembolies, les shunts intracardiaques, et en plus l’état hémodynamique [5]. Son utilisation s'est élargie au monde chirurgical pour optimiser les tactiques des réanimateurs et les techniques et résultats des chirurgiens. Son indication a été validée par des recommandations éditées par les sociétés américaines et européennes [6–8]. Dans notre pays, la demande est encore faible mais progressivement croissante, vue l'apparition récente de l'examen dans l’échiquier des examens paramédicaux dans notre contexte, et les motifs de demande sont conformes aux recommandations européennes [6]. Dans notre étude, les principales indications étaient la recherche de cardiopathies emboligènes et les endocardites.

## Conclusion

L'ETO est un examen indispensable en cardiologie. Cette étude montre son apport précieux dans le diagnostic et partant, le traitement de certaines cardiopathies. Son développement souffre malheureusement du nombre très réduit des échographistes formés et de la disponibilité des sondes. De nos jours, la formation en échocardiographie sanctionnée par le diplôme interuniversitaire n'est accessible en grande partie que dans les pays du nord. Il conviendrait dans le cadre de l'Organisation Ouest Africaine de la Santé (OOAS), qu'un diplôme sous régional soit mis en place afin de permettre au plus grand nombre d'y accéder. Ceci permettra un réel développement de l’échocardiographie en général et de l'ETO en particulier.
